# When the German Model Falters: What the 2025 Hospital Crisis Reveals About Europe's Future Healthcare System

**DOI:** 10.7759/cureus.97243

**Published:** 2025-11-19

**Authors:** Gian Marco Rizzuti

**Affiliations:** 1 Orthopaedics and Traumatology, Private Practice, Münster, DEU

**Keywords:** european health systems, germany, healthcare funding, healthcare reform, health policy, hospital crisis, hospital management, medical workforce, public healthcare, universal healthcare

## Abstract

Germany’s healthcare system, long considered a model of efficiency, is facing unprecedented strain. Economic stagnation, inflation, and funding restrictions have pushed hospitals to the edge of financial collapse, with many increasingly facing financial distress. Policy measures limiting reimbursement growth have eroded trust between hospitals and government authorities. Drawing on firsthand experience as an Italian orthopaedic surgeon working in Germany, this editorial examines how the German crisis reflects a wider European challenge. Beyond budgets, the issue lies in a cultural loss of vision, trust, and professional autonomy in public healthcare. When even the strongest system falters, the need for reform becomes urgent - not only to contain costs but to restore meaning and sustainability to medicine itself. The German case thus serves as a warning for all European health systems: without courage, competence, and long-term vision, the social contract of universal healthcare risks dissolution.

## Editorial

Germany’s economic engine is sputtering - and its hospitals are running out of breath. Once the symbol of efficiency and stability, the German healthcare system now mirrors the fragility of a continent that has forgotten how to plan for the future.

The warning signs were visible. Economic stagnation, energy costs, and growing dependence on foreign supply chains have slowed the “locomotive of Europe.” In 2025, the projected GDP growth is around 0.4% for Germany, according to the OECD [1]. The country’s industrial giants are struggling with shortages of critical components and the rising cost of labour. But the real shock comes from where Germany has always felt strongest: its hospitals.

According to the Roland Berger Krankenhausstudie 2025, three out of four hospitals ended 2024 in deficit, with nearly 89% of public facilities facing serious financial distress [2]. Staff shortages, inflation, and outdated infrastructure are pushing the system toward a structural collapse. Yet, the political response has been inconsistent - and, at times, self-defeating.

Earlier this year, the Ministry of Health limited hospitals’ ability to negotiate tariff adjustments in line with real inflation, capping reimbursement growth at 2.98%. For Gerald Gaß, president of the Deutsche Krankenhausgesellschaft, this is nothing less than Wortbruch - a breach of word [3]. The government, he argued, is undermining its own promise to link funding to the actual cost of care. The result: more closures, fewer beds, and growing disillusionment among healthcare workers.

I work inside this system. As an Italian orthopaedic surgeon in Germany, I see both the strengths and the paradoxes of this model every day. German hospitals remain highly structured and well-equipped, but they are also exhausted by bureaucracy, underfunded in core services, and increasingly disconnected from their original mission. The political debate focuses on numbers, not on people. Between spreadsheets and slogans, the link between diagnosis and therapy - both literal and metaphorical - has been lost.

The paradox is that everyone agrees reform is needed, yet every step forward seems to tighten the knot. The government has announced a €130 billion investment plan for digitalisation, infrastructure, and community care over the next five years [4]. On paper, it looks visionary. In practice, the first measure attached to it was a spending brake. Transformation requires trust and continuity - two things German healthcare no longer has.

The crisis, however, is not uniquely German. Italy offers a painful mirror. The 2025 Budget Law added €1.3 billion to the national health fund, with promises of gradual increases until 2030. But after a decade of cuts amounting to more than €37 billion, these are small repairs on a leaking dam [5]. Public hospitals remain understaffed, emergency departments are overwhelmed, and the private sector is absorbing the middle class that once relied on public medicine. The Piano Nazionale di Ripresa e Resilienza (PNRR) was supposed to modernise the system, but, regional fragmentation and political short-sightedness have turned it into another half-fulfilled promise [6].

What unites Germany and Italy - and perhaps Europe as a whole - is not only the financial pressure but a deeper cultural fatigue. Healthcare has become a technical problem instead of a social contract. Ministers speak the language of “efficiency,” but avoid the words “solidarity” and “trust.” Doctors are asked to do more with less, while patients are told to expect less and pay more. Somewhere along the way, we replaced vision with management.

The German debate, at least, remains brutally honest. Hospital executives, professional associations, and even journalists openly challenge the government’s logic [7]. In Italy, discussion is often reduced to slogans or emergencies. Yet both countries share the same disease: short-term politics, reactive governance, and the loss of strategic imagination.

Beyond the national contrasts, Europe is facing a common dilemma: demographic ageing, workforce burnout, and a shrinking pool of trained physicians. The WHO and OECD warn that by 2030, Europe could face a shortage of more than four million healthcare workers [8,9]. Germany already reports over 35,000 vacant nursing positions and a deficit of 15,000 physicians [10]. When hospital wards close not for lack of patients but for lack of staff, the issue transcends economics - it becomes moral and existential.

If even Germany - the benchmark of European stability - falters, then no system is immune. What we are witnessing is not just an economic downturn, but the erosion of a shared idea of public health. A continent that once built its identity on universal healthcare is now negotiating its survival within budget spreadsheets.

Rebuilding trust in medicine will take more than digitalisation or cost containment. It will require a new narrative - one that puts competence before rhetoric and courage before convenience. For Europe’s hospitals, this is not merely a time for reforms. It is a time for honesty, vision, and moral reconstruction.
